# *Epilepsia Open* – April 2025 Announcements

**DOI:** 10.1002/epi4.70025

**Published:** 2025-04-22

## ILAE CONGRESSES

7th ILAE School on EEG in the First Year of Life

26 ‐ 27 April 2025

Sanya City, Hainan, China

ILAE School on Neuroimaging 2025

15 ‐ 17 May 2025

Potsdam, Germany

5th African Epilepsy Congress

16 ‐ 18 May 2025

Lusaka, Zambia

Curso‐taller internacional de electroencefalografia básica y avanzada

23 ‐ 24 May 2025

En línea

15th ILAE School for Neuropathology and Neuroimaging in Epilepsy

31 July ‐ 3 August 2025

Campinas, São Paulo, Brazil

XVIII Workshop on Neurobiology of Epilepsy (WONOEP 2025)

25 ‐ 29 August 2025

Portugal

36th International Epilepsy Congress

30 August ‐ 3 September 2025

Lisbon, Portugal

## 2026

15th ILAE School on Pre‐Surgical Evaluation for Epilepsy and Epilepsy Surgery

19 ‐ 23 January 2026

Brno, Czech Republic

16th European Epilepsy Congress

5 ‐ 9 September 2026

Athens, Greece

## ILAE WEBINARS

Epilepsy and Stroke

1 April 2025

Crises occipitales et pariétales

8 April 2025

Focal Ictogenesis: When and why does interictal activity transition into ictal activity?

10 April 2025

ILAE e‐Forum: Diagnosis and classification of Developmental and Epileptic Encephalopathies (DEE)

22 April 2025

Thalamo‐cortical Network: How does coupling between focus and thalamus change before seizures?

24 April 2025

ILAE Eastern Mediterranean Webinar

24 April 2025

Neurodevelopment

1 May 2025

SUDEP: What are the mechanisms and risk factors?

13 May 2025

Neurodegeneration: When and how do neurodegenerative diseases lead to seizures?

20 May 2025

Understanding Functional Seizures in Children

17 June 2025

## OTHER CONGRESSES

AD/PD™ 2025 International Conference on Alzheimer's and Parkinson's Diseases and related neurological disorders

1 ‐ 5 April 2025

Vienna, Austria & Online

International Congress on Structural Epilepsy & Symptomatic Seizures 2025

2 ‐ 4 April 2025

Gothenburg, Sweden

EPIPED Course: Treatment Strategies in Pediatric Epilepsies

23 ‐ 26 April 2025

Girona, Spain

12th International Residential Course on Drug Resistant Epilepsies

11 ‐ 27 May 2025

Tagliacozzo, Italy

International Conference for Technology and Analysis of Seizures (ICTALS 2025)

2 ‐ 6 June 2025

Montreal, Canada

The Comorbidities of Epilepsy Course

20 June 2025

London, UK & Online

Korean Epilepsy Congress 2025

20 ‐ 21 June 2025

Seoul, South Korea

11th Congress of the European Academy of Neurology

21 ‐ 25 June 2025

Helsinki, Finland

11th Combined Migrating Caucasian Course on Epileptology (CMCCE)

3 ‐ 6 July 2025

Tbilisi, Georgia

21st San Servolo Advanced Epilepsy Course

21 July ‐ 1 August 2025

San Servolo (Venice), Italy

5th International Congress on Mobile Health and Digital Technology in Epilepsy

4 ‐ 6 September 2025

Copenhagen, Denmark

19th European Congress for Clinical Neurophysiology

9 ‐ 12 September 2025

London, UK

ILAE British Young Epilepsy Section (YES) Early Career Building Day

14 September 2025

Bournemouth, UK

ILAE British Branch 2025 Annual Scientific Conference

15 ‐ 17 September 2025

Bournemouth, UK

19th Panhellenic Congress of Epilepsy

25 ‐ 28 September 2025

Heraklion, Crete, Greece

The 2025 ILAE British Branch Clinical Epilepsy 1‐Day Course for Doctors in Training & Others

22 November 2025

Birmingham, UK

## 2026

18th Eilat Conference on New Antiepileptic Drugs and Devices

3 ‐ 6 May 2026

Madrid, Spain

